# Characterization of Dystrophin Deficient Rats: A New Model for Duchenne Muscular Dystrophy

**DOI:** 10.1371/journal.pone.0110371

**Published:** 2014-10-13

**Authors:** Thibaut Larcher, Aude Lafoux, Laurent Tesson, Séverine Remy, Virginie Thepenier, Virginie François, Caroline Le Guiner, Helicia Goubin, Maéva Dutilleul, Lydie Guigand, Gilles Toumaniantz, Anne De Cian, Charlotte Boix, Jean-Baptiste Renaud, Yan Cherel, Carine Giovannangeli, Jean-Paul Concordet, Ignacio Anegon, Corinne Huchet

**Affiliations:** 1 INRA, UMR703 APEX, Oniris, Atlantic Gene Therapies, Université de Nantes, Oniris, École nationale vétérinaire, agro-alimentaire et de l'alimentation, Nantes, France; 2 INSERM, UMR 1087/CNRS 6291 Institut du Thorax, Université de Nantes, Faculté des Sciences et des Techniques, Nantes, France; 3 INSERM, UMR 1064-Center for Research in Transplantation and Immunology, ITUN, CHU Nantes, Université de Nantes, Faculté de Médecine, Nantes, France; 4 INSERM, UMR 1089, Atlantic Gene Therapies, Thérapie génique pour les maladies de la rétine et les maladies neuromusculaires, Université de Nantes, Faculté de Médecine, Nantes, France; 5 Genethon, Evry, France; 6 INSERM, U1154, CNRS, UMR 7196, Muséum National d’Histoire Naturelle, Paris, France; University of Minnesota Medical School, United States of America

## Abstract

A few animal models of Duchenne muscular dystrophy (DMD) are available, large ones such as pigs or dogs being expensive and difficult to handle. *Mdx* (*X-linked muscular dystrophy*) mice only partially mimic the human disease, with limited chronic muscular lesions and muscle weakness. Their small size also imposes limitations on analyses. A rat model could represent a useful alternative since rats are small animals but 10 times bigger than mice and could better reflect the lesions and functional abnormalities observed in DMD patients. Two lines of *Dmd* mutated-rats (*Dmd^mdx^*) were generated using TALENs targeting exon 23. Muscles of animals of both lines showed undetectable levels of dystrophin by western blot and less than 5% of dystrophin positive fibers by immunohistochemistry. At 3 months, limb and diaphragm muscles from *Dmd^mdx^* rats displayed severe necrosis and regeneration. At 7 months, these muscles also showed severe fibrosis and some adipose tissue infiltration. *Dmd^mdx^* rats showed significant reduction in muscle strength and a decrease in spontaneous motor activity. Furthermore, heart morphology was indicative of dilated cardiomyopathy associated histologically with necrotic and fibrotic changes. Echocardiography showed significant concentric remodeling and alteration of diastolic function. In conclusion, *Dmd^mdx^* rats represent a new faithful small animal model of DMD.

## Introduction

DMD is a severe X-linked muscular dystrophy due to mutations of the *DMD* gene. It affects all voluntary muscles as well as the heart and breathing muscles in later stages. Despite recent promising new treatments the average life expectancy is severely reduced in DMD patients and a better understanding of the disease and faster evaluation of new treatments are needed [1].

Both large and small animal species deficient for dystrophin have been described and have been extremely useful for pre-clinical studies of DMD. Although they display more features of the human clinical phenotype than *mdx* mice, large dystrophin*-*deficient animals such as dogs [2] and pigs [3], [4], suffer from individual variability and are costly and time consuming. *Mdx* mice [5], [6] have the advantage of low maintenance costs. Sufficient numbers of animals can also be easily characterized to reach high statistical power. On the other hand, *mdx* mice exhibit only minor clinical dysfunction [6] and their small size imposes limitations in the analysis of several aspects of the disease. Although each animal model has its own limitations, they have all been essential for the development of treatment strategies that target dystrophin absence, disease progression or muscle regeneration [7]. Nevertheless, new animal models are needed to help pre-clinical research on DMD.

We hypothesized that the rat could represent a useful model of DMD. One of its advantages of over mice is that its behavior is much better characterized. Rats have finer and more accurate motor coordination than mice and exhibit a richer behavioral display, including more complex social traits [8]. Rats have a convenient size since they are 10 times larger than mouse but are still a small laboratory animal model and allow studies with high statistical power. Until recently, the rat model lacked genetic engineering tools for introducing targeted genetic mutations. But in the last years, we and others have used in rats sequence-specific nucleases, such as meganucleases, zinc-finger nucleases, TALENs and CRISPRs/Cas9, to efficiently generate precise gene mutations [9]–[13].

To generate dystrophin*-*deficient rats, we generated TALENs for *Dmd* that were microinjected in rat zygotes allowing generation of two *Dmd^mdx^* rat lines. The muscles of both lines displayed undetectable levels of dystrophin as evaluated by western blot analysis and less than 5% of dystrophin positive fibers by immunohistochemistry. At 3 months of age, forelimb, hindlimb and diaphragm muscles showed severe fiber necrosis and a strong regeneration activity. At 7 months of age, regeneration activity was decreased and muscle showed abundant peri- and endomysial fibrosis with some adipose tissue infiltration. Muscle strength and spontaneous activity were decreased and fatigue was a prominent finding of muscle function analysis. Cardiac muscle was also affected with necrosis and fibrosis and showed signs of progressive dilated cardiomyopathy. Echocardiography showed significant concentric remodeling and alteration of diastolic function. These lesions in skeletal muscle and heart closely mimic those observed in DMD patients. These results indicate that *Dmd^mdx^* rats represent a new invaluable small animal model for pre-clinical research on DMD.

## Materials and Methods

### Animals

This study was approved by the Ethics Committee on Animal Experimentation of the Pays de la Loire Region, France, in accordance with the guidelines from the French National Research Council for the Care and Use of Laboratory Animals (Permit Numbers: CEEA-PdL-2011-45 and CEEA-PdL-01579.01). All efforts were made to minimize suffering. Sprague-Dawley (SD/Crl) rats were obtained from Charles River (L’Arbresle, France). The rats were housed in a controlled environment (temperature 21±1°C, 12-h light/dark cycle). Before blood collection, animals were anesthetized with a mixture of ketamine (100 mg/kg, Imalgene, Merial, Lyon, France) and xylazine (10 mg/kg, Rompun, Bayer, Leverkusen, Germany). Rats were then sacrificed by intravenous administration of sodium pentobarbital (300 mg, Dolethal, Vetoquinol UK Ltd, Buckingham, UK). Just after sacrifice, the body weight (g) and the body length (cm) of each rat were determined to define the body mass index calculated as body weight/(body length)^2^ (g/cm^2^).

### Design and production of TALE nucleases

TALE nucleases targeting *Dmd* exon 23 were designed to recognize the following sites on Rat genome Assembly (Rnor_5.0 -GCA_000001895.3/chrX: 51,878,333-52,510,293).

TCTGCAAAGCTCTTTGAAA GAGCAACAAAATGGCTTCAACTATCTGAATGCCA .

TALE nucleases were produced, as previously described, by unit assembly method adapted from Huang et al. 2011 [14]–[16]. For each TALE nuclease subunit, the fragment containing the 16 RVD segment was obtained from single unit vectors: A (NI), T (NG), G (NN) and C (HD), derived from plasmids kindly provided by the laboratory of Dr B. Zhang (Peking University, China). The assembled TALE RVD (DMD-L: TCTGCAAAGCTCTTTGAAA; DMD-R: TGGCATTCAGATAGTTGA ) were subcloned in the pVax vector containing the 17th half RVD, the Δ152/+63 N- and C-terminal truncation points as described by Miller et al. 2011 [17] and contained the wt Fok I catalytic domains or FokI heterodimer-forcing mutations (ELD or KKR) [18] that were constructed by site-directed mutagenesis starting from Addgene plasmids 21872 et 21873 kindly made available by the Joung lab. Assembled TALE nucleases DNAs were cloned into the pVAX vector for mRNA production and *in* *vitro* cell transfection.

### *In vitro* assay of TALE nucleases

Each subunit of TALE nuclease (0.75 and 1.5 µg each) was nucleofected into 4.10^5^ C6 cells (Sigma) in 20 µL of V solution using AMAXA FF137 program. Cells were grown into 12 well dishes and collected 48 h after nucleofection. Genomic DNA was extracted with E.Z.N.A. Tissue DNA Kit (OMEGA Biotek). The genomic region encompassing the TALE nuclease target sites was amplified with the primers listed below, typically using 50 ng of genomic DNA, and phusion polymerase (New England Biolabs) following manufacturer instructions. Unpurified PCR product (10 µL out of 25 µL of PCR reaction) was supplemented with 10 µL of NEBuffer 2 (2X), melted, and annealed (5 min at 95°C, 95°C to 25°C at −0.5°C/30 sec, and 15 min at 4°C) to form heteroduplex DNA. The annealed DNA was treated with 1.5 units of T7 endonuclease I (New England BioLabs) for 20 min at 37°C and run on a 2.4% agarose gel.

### Off-target mutation analysis

Potential off-target sites were identified by bioinformatics using the PROGNOS program [19] with parameters set for heterodimer-forcing TALEN subunits and looking for pairs of potential TALEN binding sequences with up to 6 mismatches to each cognate target sequences of the TALEN pair separated by spacer of 10 to 30 bp. Candidate off-target sites that were identified had at least a total of 8 mismatches to the TALEN target sequence in the DMD gene. We chose to further look for mutations at the 2 candidate sites lying on the X-chromosome because they could potentially cosegregate with the DMD mutations and affect the phenotype of the rats investigated. The candidate sites found on X-chromosomes were TttCATTCAGcTAtTTGAAATGGGAAGACAGCACACTGATCCATTTttAAGAGgTTTGCAGc and TCTatAAcGCcCTTTaAAAATGGAATAAGATCCTTTGCAAGTGATTtAcCTATaTGAATGtgA with potential TALEN binding sites underlined and lowercase indicating mismatches to the TALEN DMD target sequence. The presence of mutations was tested using the T7 endonuclease E1 assay as detailed above using specific primers for PCR listed below. No mutations could be detected at candidate off-target sites from the X-chromosome in tail DNA from 6 F0 (including the founder rats #61 and #71) and 8 F2 rats derived from founder rat #61 (including 4 DMD mutant animals) using the T7 endonuclease 1 assay (data not shown).

Primer sequences:

DMDs CAAGTATGCATCGGTTAGTGTA
DMDas GCATCAATAACTTTGAGGGACT
XoffN1s CGAAATAGAGCTAGAATCCCCAGG
XoffN1as GTT TCC AAG GGA CAG ACA ACA CAG
XoffN2s CAG TCC TCT TTG CTC ATG TGC TAG
XoffN2as CCC TTG TGT GTG TGT GTG TGT ATG

### *In vitro* transcription of TALE nucleases mRNA

As previously described, TALE nuclease plasmids were *in* *vitro* transcribed to mRNA and polyadenylated using the mMessage mMachine T7 Ultra kit (Ambion, Austin, TX) following the manufacturer protocol and purified using the MegaClear Kit (Ambion, Austin, TX), quantitated using a NanoDrop-1000 (Thermo Scientific) and stored at −80°C until use [12]. mRNAs encoding each monomer of TALE nucleases for each target sequence were mixed in TE 5/0.1 (5 mM Tris-Cl pH 7.5, 0.1 mM EDTA in RNase DNase free water) and stored at −80°C until use. mRNAs were diluted to the working concentration and kept on ice during one day micro-injection procedures and then discarded.

### Microinjection of rat one-cell embryos

Prepubescent females (4–5 weeks old) were super-ovulated with pregnant mare serum gonadotropin (30 IU; Intervet, France) and followed 48 hours later with human chorionic gonadotropin (20 IU; Intervet, France) before breeding. Zygotes were collected for subsequent microinjection using a previously published procedure [12], [20]. Briefly, a mixture of TALE nucleases mRNA was microinjected into the cytoplasm of fertilized one-cell stage embryos. Microinjected embryos were maintained under 5% CO_2_ at 37°C until reimplantation. Surviving embryos were then implanted immediately in the oviduct of pseudo-pregnant females (0.5 dpc) and allowed to develop until term.

### Analysis of *Dmd* mutation events

DNA from embryos or neonates was extracted from tail biopsy following treatment with Proteinase K as previously described [12]. To analyze mutations, we first used PCR followed by the T7 endonuclease I assay (as described above in the *in* *vitro* analysis of mutations) to identify rats carrying mutations and PCR products were sequenced.

### Dystrophin messenger analysis

Total RNA was extracted from muscles with TRIzol reagent (Invitrogen) and 500 ng of this RNA was reverse transcribed using random primers (Invitrogen) and M-MLV reverse transcriptase (Invitrogen). Detection of the Dystrophin mRNA was done by 25 cycles of PCR1 followed by 30 cycles of PCR2 using 1 µl of ADNc or 1 µl of PCR1, 2,5 U of GoTaq DNA polymerase (Promega), 1,5 mM of MgCl2 and 0,2 µM of each following primers (PCR1: ex20ratDyst-F TCA GAC AAG CCT CAG AAC AA and ex26ratDyst-R AGTTTTATCCAAACCAGCCT annealing 50°C; PCR2:ex22ratDyst-F AAT GCG CTA TCA AGA GAC AA and ex24ratDyst-R TCT GCA CTG TTT GAG CTG TT annealing 55°C). Final PCR products were migrated on an 1.5% agarose gel and revealed with ethidium bromide staining (**Fig. S6**).

### Western blot analysis

Total proteins from muscle were extracted using 400 µL of RIPA extraction buffer containing protease inhibitors (Roche) and ground with TissueLyser II (Qiagen). 50 µg of protein extracts were loaded on a 3–8% Tris-Acetate Precast polyacrylamide gel of NuPAGE Large Protein blotting kit (Invitrogen). After Red Ponceau staining, membranes were incubated with two different mouse anti-Dystrophin antibodies: NCL-DYS2 (1∶100, Novocastra), MANEX 1011C (1∶100, MDA Monoclonal Antibody Resource) [21] and with an anti-GAPDH antibody (1∶10000, Imgenex). Detection was performed using a secondary anti-mouse IgG HRP-conjugated antibody (1∶2000, Dako) or secondary anti-goat IgG HRP-conjugated antibody (1∶2000, Dako). Immunoblots were revealed with ECL Western blotting substrate (Pierce) and exposed to ECL-Hyperfilm (Amersham).

### Histopathological evaluation

After gross examination, *tibialis cranialis, extensor digitorum longus, biceps femoris* muscles of the hindlimb, *soleus* and *biceps brachii* muscles of the forelimb, diaphragm and heart were sampled and divided in two parts. Furthermore, *tibialis cranialis, extensor digitorum longus, soleus* and heart were weighted. The first part was frozen and 8-µm-thick serial sections were cut for immunohistology and histoenzymology assays, the second part was fixed in 10% neutral buffered formalin, embedded in paraffin wax, and 5-µm-thick sections were cut and routinely stained with hematoxylin eosin saffron for histopathological evaluation. Additional sections were stained with Picrosirius red stain for collagen, Gomori's trichrome and Alizarin red staining for calcium. All lesions were reported by a skilled pathologist certified by the European College of Veterinary Pathology.

### Immunohistochemistry and histoenzymology assays

Immunohistochemical analysis involved the use of immunoperoxidase techniques on frozen sections. MANDYS110 clone 3H10, provided by the Wolfson Centre for Inherited Neuromuscular Disease, NCL-DYSB and NCL-DYS2 (Novocastra Laboratories, Newcastle on Tyne, UK) mouse monoclonal antibodies were used for dystrophin protein detection (1∶50 for each ones). Other primary antibodies used were IVD3_1_A9 (1∶50, Developmental Studies Hybridoma Bank, Iowa City, IA), NCL-g-Sarc (1∶10, Novocastra Laboratories) and NCL-DRP2 (1∶60, Novocastra Laboratories) for alpha-sarcoglycan, gamma-sarcoglycan, and utrophin detection respectively, NCL-MHCd (1∶20, Novocastra Laboratories) for developmental myosin heavy chain isoform, anti-complement 5b-9 (1∶250, Calbiochem, Strasbourg, France) and anti-CD3 (1∶100, Dako, Glostrup, Denmark) for lymphocytes. Briefly, transverse cryosections were incubated in PBS with 5% normal goat serum (Dako) for 1 hour at room temperature. They were then incubated with primary antibody in 5% rat serum overnight at 4°C and with biotinylated secondary antibodies (E433, 1∶300, Dako) in PBS with 5% rat serum for 1 hour. Bound antibodies were detected either with streptavidin (P397; Dako) and DAB Liquid Substrate (Dako) for immunoperoxidase. Fiber type was determined using histochemical myosin-ATPase reaction after preincubation at pH 4.2, 4.35, and 10.4 as previously described [22].

### Histomorphometry

Morphometric analysis was done using a digital camera (Nikon DXM 1200; Nikon Instruments, Badhoevedorp, the Netherlands) combined with image-analysis software (NIS; Nikon). To determine the proportion of dystrophin-positive fibers in the *biceps femoris* muscle of 3 and 7 month-old *Dmd^mdx^* rats (5 individuals in each group), a total of 1,000 fibers were counted in sections immunolabelled with Mandys110 antibody and the percentage of fibers expressing dystrophin was determined. To determine the percentage of MyoHC_Dev_ fibers, at least 1,000 fibers were numbered on randomly selected microscopic fields. Fiber size was documented measuring minimal Ferret diameter on at least 250 fibers (292±61 per sample), on randomly selected microscopic fields in picrosirius red-stained sections. Fibrosis was determined as the ratio of areas rich in collagen using specific picrosirius staining on the total muscle area in an overall cross section. Repeatability was tested by the same operator measuring five times the same sample. The intra-assay variations coefficients were always lower than 5%.

### Serum creatine phosphokinase levels

Blood was collected just before sacrifice under anesthesia. Creatine phosphokinase (CK) activity was determined at the Central Laboratory of the National Veterinary School (E.N.V) at Nantes using a commercial kit (LaRoche Diagnostic). Results were expressed as CK levels [23].

### Skeletal muscle function

The *in* *vivo* tests were performed in the same sequence for each rat, with equivalent time of rests between the tests [23].

#### Grip test

Rats were placed with their forepaws or four paws on a grid and were gently pulled backward until they released their grip [24]. A grip meter (Bio-GT3, BIOSEB, France), attached to a force transducer, measured the peak force generated. Five tests were performed in sequence with a short latency between each test, and the reduction in strength between the first and the last determination was taken as an index of fatigue [24]. Results are expressed in grams (g) and normalized to the body weight (g/g).

#### Actimeter

The motor behavior was examined with an open field actimeter [25]. For this analysis, rats were individually placed in an automated photocell activity chamber (Letica model LE 8811, Bioseb, France) which consists of a plexiglass chamber (45 cm×45 cm×50 cm) surrounded by two rows of infrared photobeams. The first row of sensors was raised at a height of 3 cm for measuring horizontal activity and the second row placed above the animal for vertical activity. The spontaneous motor activity was measured for 5 min using a movement analysis system (Bioseb, France), which dissociates resting and ambulatory time (s), distance traveled (cm), stereotyped, and rearing movements.

### Echocardiography

Two-dimensional (2-D) echocardiography was performed on rats using a MyLab70 Family - Ultrasound Systems – Esaote with a CA129 transducer [26]. In order to look for possible structural remodelling, the left ventricular end-diastolic diameter and the free wall end-diastolic thickness were measured during the diastole from long and short-axis images obtained by M-mode echocardiography. Furthermore, the systolic function was assessed by the ejection fraction, while transmitral flow measurements of ventricular filling velocity were obtained using pulsed Doppler, with an apical four-chamber orientation. Thus, Doppler–derived mitral deceleration time, the early diastolic (E), the late diastolic (A) and the ratio E/A were obtained to assess diastolic dysfunction associated to an evaluation of the isovolumetric relaxation time. To avoid bias in the analysis, experiments were done in a blind fashion.

### Statistics

Differences between means were analyzed by unpaired Student's *t*-test. When the Student test was not applicable a Mann Whitney test was used. All analyses were performed using SigmaStat 3.1 software (Logi Labo, Paris, France) and Xlstat (Addinsoft, Paris, France). The data are presented as mean ± S.E.M. with the significance level set at *p*<0.05.

## Results

### Generation of *Dmd* mutated rats

We generated a pair of TALENs targeting exon 23 of the rat *Dmd* gene in order to generate rats with *Dmd* mutations comparable to that of the *mdx* mouse (**Fig. S1**) [11], [14]. The TALE DNA-binding domains were fused either to the wild-type FokI nuclease domain that can form homodimers or to mutated forms (so called ELD or KKR mutants for each monomer) that can only form heterodimers and thus will have less potential off-target effects. Plasmids encoding wild-type or ELD/KKR TALENs were transfected into rat C6 cells at different doses and were found to induce mutations in dose dependent fashion as evidenced by the PCR-T7 endonuclease I assay (**Fig. S1**). Even if that the wild-type pair was more effective at inducing mutations, we chose to use the heterodimeric ELD/KKR TALEN pair for the sake of improved specificity.

The mRNAs of each ELD/KKR TALEN monomer were microinjected into the cytoplasm of 387 rat zygotes, 320 were viable and 294 were transferred into pseudopregnant females, as previously described (**Table S1**) [11], [12]. This resulted in the 88 newborns and among them 11 showed mutations (3.74% of the transferred zygotes and 12.5% of newborns). These results are similar to our previous ones injecting other engineered sequence-specific nucleases and thus *Dmd* TALENs did not trigger embryo toxicity and allowed to induce *Dmd* gene mutations as expected [9]–[12].

**Figure 1:**
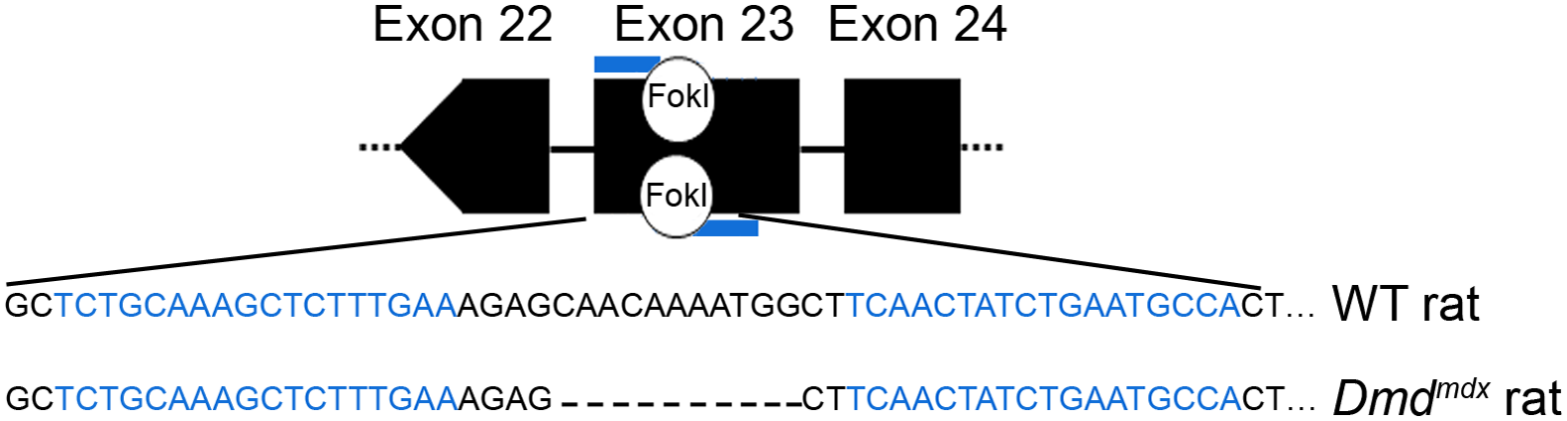
Targeted deletion in exon 23 of the rat *Dmd* gene via TALE nucleases. DNA cleavage takes place in the spacer sequence located between TALEN DNA binding sequences indicated in blue. *Dmd^mdx^* rats carries a deletion of 11 nucleotides in exon 23 of the *Dmd* gene.

The *Dmd* mutations observed in newborn rats were deletions ranging from 1 to 24 bp and insertions of a few nucleotides (**Table S2**). Two of the newborns presented mutations that did not disrupt the open reading frame and were not further analyzed. In the other newborns, the mutations disrupted the open reading frame and resulted in premature stop codons. Some animals with *Dmd* mutations detected in tail DNA did not transmit the mutation to their offspring, most likely because it was not present in the germ line due to late activity of TALENs after injection, while several of them transmitted the mutations to the offspring in a Mendelian manner: two of these rat lines were further analyzed (lines 61 and 71). Both lines displayed the same muscle anatomopathological and functional characteristics and therefore only line 61, hereafter called *Dmd* ^mdx^ is presented in this report. *Dmd* ^mdx^ mutation is a deletion of 11 bp in exon 23 leading to a +1 frame shift and premature stop codon 81 bp after the mutation (**Fig. 1** and **Table S2**).

Female offspring of mutant founders that carried the mutation did not show premature death and were indistinguishable from wild-type littermate controls (**Fig. S2 *A***). X-linked DMD mainly affects boys and we have therefore only characterized male *Dmd^mdx^* rats in this study. When compared to wild-type littermate controls of the same ages, *Dmd^mdx^* rats are smaller and less heavy (**Table 1** and **Fig. S2 *B***). As illustrated by the increase in weight with age, the growth of *Dmd^mdx^* rats is significantly altered as soon as 4 weeks of development (**Fig. S2 *C***).

**Table 1:** Rat weight and size as well as isolated muscles weight and heart characteristics of *Dmd^mdx^* male rats.

Age	3 month-old		7 month-old	
	WT	*Dmd^mdx^*	WT	*Dmd^mdx^*
***Rat characteristics***				
Body weight (g)	456.0±21.2	409.4±18.9	749.7±45.9	501.6±30.1*
Body length (cm)	25.9±0.4	25.5±0.3	28.9±0.4	27.1±0.4*
Body mass index (g/cm^2^)	0.68±0.02	0.63±0.02	0.89±0.03	0.68±0.03*
**Muscle weight**				
*Tibialis Anterior* (mg)	751.8±41.2	822.9±50.2	1009.2±57.1	773.1±58.1*
*Tibialis Anterior* (mg/g)	1.65±0.02	2.00±0.07	1.35±0.07	1. 54±0.06*
*Extensor Digitorum Longus* (mg)	200.6±10.4	205.0±10.5	257.2±14.0	193.2±15.9
*Extensor Digitorum Longus* (mg/g)	0.44±0.01	0.50±0.01	0.34±0.02	0.38±0.01
* Soleus* (mg)	184.9±6.5	199.9±10.8	206.2±8.2	189.0±13.7
* Soleus* (mg/g)	0.41±0.01	0.49±0.02	0.28±0.01	0.38±0.02
**Heart characteristics**				
Weight (mg)	1379.5±84.7	1732.2±271.1	2049.8±72.6	1766.3±84.4*
Weight (mg/g)	3.02±0.13	4.21±0.58	2.75±0.11	3.55±0.16*
p/LV (%)	56.0±0.1	44.9±0.03 §	43.1±1.8	35.8±3.9 §
*n*	*5*	*5*	*4*	*5*

body mass index calculated as body weight/(body length)^2^; (g/cm^2^).

Values are means ± SEM; *: p<0.05 *vs* wild-type controls (WT); §: p<0.0001 *vs* WT. n, numbers of rats.

Founder 61 and its offspring were analyzed for potential off target mutations at other loci. The closest homologous sequences to the ones recognized by *Dmd* TALENs showed eight mismatches. We chose to further look for mutations at the 2 candidate sites located on the X-chromosome because they could potentially cosegregate with the *Dmd* mutations and affect the mutant rat phenotype (**Table S2**). They were analyzed no mutations could be detected by T7 endonuclease I assays (data not shown). Altogether, these results indicate that rat lines with a specific disruption of the *Dmd* coding frame by premature stop codons were successfully generated.

### Analysis of dystrophin expression

**Figure 2:**
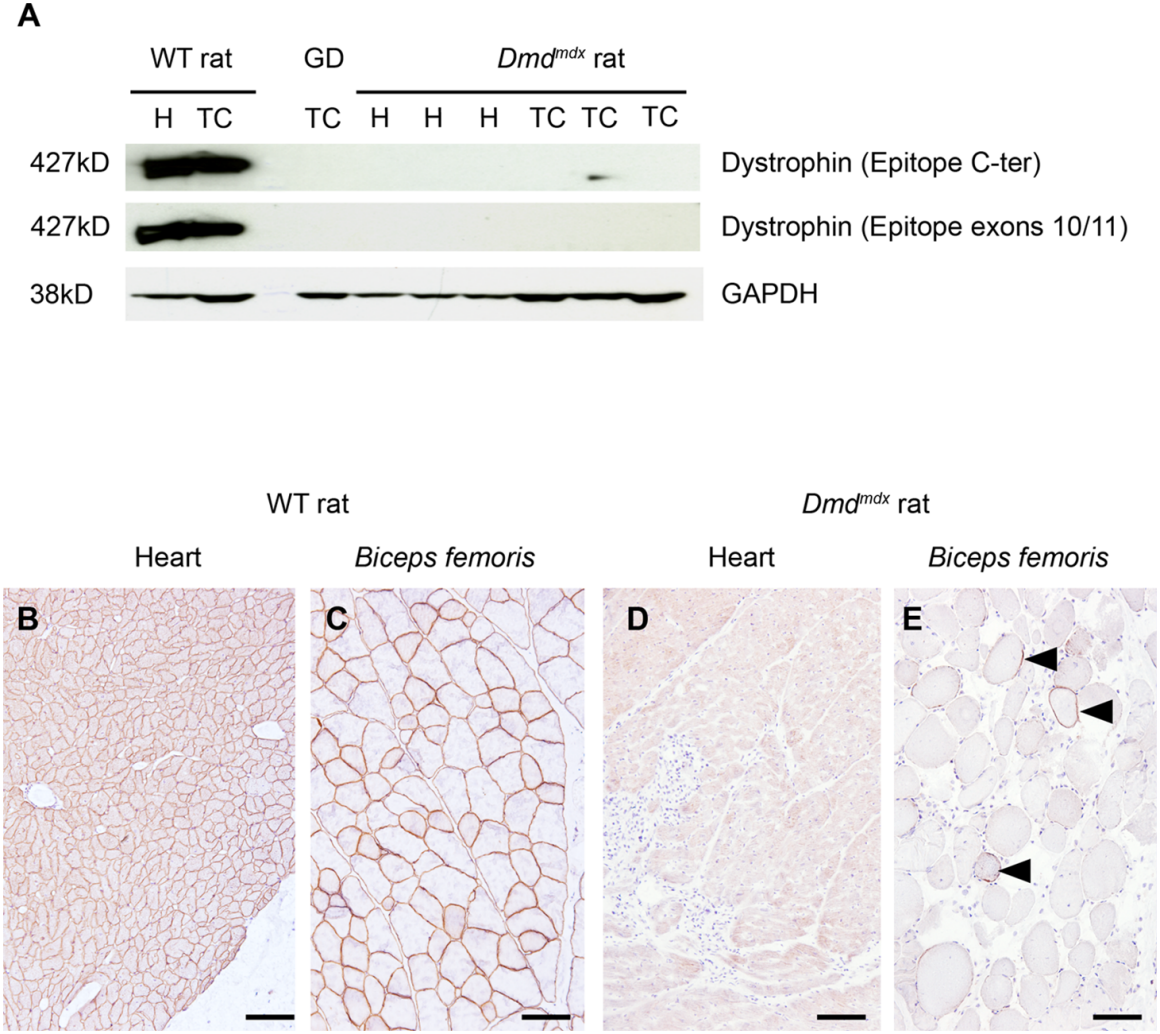
No dystrophin expression was detected in cardiac and skeletal muscles of *Dmd^mdx^* rats. (*A*) Male 7 month-old rats of line 61, wild-type littermate controls (WT) and *Dmd^mdx^* were sacrificed and biopsies from *tibialis cranialis* muscles (TC) and hearts (H) were harvested. Western-blot of total proteins (50 µg) was incubated with NCL-DYS2 and Manex1011C monoclonal antibodies (C-terminal and exons 10/11 epitopes, respectively). This revealed undetectable levels of the 427 kDa dystrophin band in line 61 *Dmd^mdx^* rats. Muscle from a GRMD dog (GD) was used as negative control and samples from WT rats were used as positive controls. Staining with an anti-GAPDH polyclonal antibody validated equal protein loadings. (*B–E*) Heart and *biceps femoris* muscles from the same wild-type (*B* and *C*) and *Dmd^mdx^* rats (*D* and *E*) were assessed for dystrophin expression using immunohistochemistry with Mandys110 monoclonal antibody (against exons 38–39 epitope). Compared to the subsarcolemmal expression of dystrophin in wild-type muscles, no dystrophin was detected in *Dmd^mdx^* rats except for the presence in skeletal muscle of only rare scattered revertant positive fibers (arrowheads). Immunolabelling of dystrophin (*B–E*) Bar = 100 µm.

Western blot analysis of dystrophin expression in *tibialis cranialis* and cardiac muscles was performed using two antibodies directed against epitopes located at the C terminus and within exons 10–11. No expression of dystrophin or of a truncated dystrophin could be detected in muscle tissues analyzed from *Dmd* mutated animals (**Fig. 2** ***A***).

Immunohistological analysis of *biceps femoris* and cardiac muscles using mouse monoclonal antibodies directed against three different epitopes (located within exons 11–12, 38–39 and exon 78) revealed rare scattered dystrophin positive fibers in all *Dmd* mutated animals (**Fig. 2** ***B*** **–** ***E*** for epitope within exons 38–39 and data not shown). Dystrophin positive fibers in *biceps femoris* of mutated animal of line 61 after immunolabelling represented 4.9±0.8% (n = 5) of total number of fibers. Such dystrophin positive fibers are frequently observed in animal models of dystrophin deficiency [26]. The exact mechanism involved in the generation of the so-called revertant myofibers remains poorly understood.

The dystrophin-associated protein complex is severely affected in skeletal muscle of DMD patients. We therefore examined the expression of alpha- and gamma- sarcoglycan, two proteins of the dystrophin-associated complex. The expression of alpha-sarcoglycan (**Fig. S3 *A, B***) and gamma (data not shown) was weak and scarce compared to the systematic expression in myofibers of wild- type rats [27]. Altogether, these results indicate that *Dmd* ^mdx^ rats are *bona fide* dystrophin deficient animals.

### Histopathological evaluation of muscles

Before histopathological evaluation, skeletal muscle and heart weights were measured in 3 and 7 month-old rats. Fast muscles *tibialis anterior* and *extensor digitorum longus* muscles reached significantly lower weights, of around 25% less, in 7 month-old *Dmd^mdx^* rats compared to wild-type controls (**Table 1**). In contrast, the slow *soleus* skeletal muscle was not significantly affected. The loss of skeletal muscle mass that was detected is consistent with the lower body weight of *Dmd^mdx^* rats (**Fig. S2 *C***) and indicates that muscular atrophy takes place in *Dmd^mdx^* rats, preferentially in fast muscles. The heart weights were slightly higher at 3 months and significantly lower at 7 months in *Dmd^mdx^* rats compared to littermates (**Table 1**). At 3 months, hearts of *Dmd^mdx^* rats were markedly dilated with left ventricular wall thinning and increased diameter of the ventricular cavity. Ratio between left ventricle wall thickness and ventricular cavity diameter was significantly decreased compared to age-related wild-type littermate controls at 3 and 7 months (**Table 1**). All together these changes in heart morphology were indicative of a progressive dilated cardiomyopathy in *Dmd^mdx^* rats.

**Figure 3:**
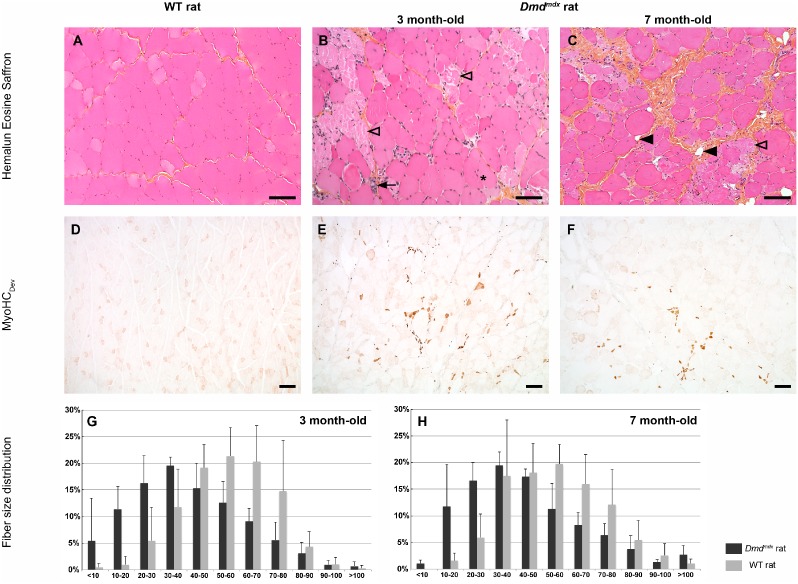
Severe and progressive muscle changes in *Dmd^mdx^* rats. *Biceps femoris* muscle was sampled at 3 and 7 month-old from wild-type littermate controls (WT) and *Dmd^mdx^* rats. Compared to controls (*A*), 3 month-old *Dmd^mdx^* rat skeletal muscles (*B*) displayed individual fiber necrosis (open arrowhead) associated with foci of small newly regenerating centronucleated fibers (arrow). In addition, 7 month-old muscles displayed a progessive replacement of fibers by fibrosis (*) and fat (black arrowhead) tissue (*C*). The regenerative activity was assessed using a specific antibody against the Myosin Heavy Chain developmental isoform (MyoHC_Dev_). No regenerative activity was observed in control rats (*D*) whereas newly regenerating fibers were numerous in *Dmd^mdx^* rats (*E*) with a decrease in their number with age (*F*). Histological changes were quantified. Fiber minimal Ferret diameter was measured. A global switch towards lower sized fibers was observed in the fiber size distribution in mutated rats compared to controls at 3 and 7 month of age (*G–H*). Hemalun eosin saffron staining (*A–C*), immunolabelling of Myosin Heavy Chain developmental isoform (MyoHC_Dev_) (*D–F*). Bar = 100 µm (*A–C*) and 200 µm (*D–F*).

Histopathological evaluation of *tibialis cranialis, extensor digitorum longus, biceps femoris, biceps brachii, soleus* and diaphragm skeletal muscles and heart was performed at 3 and 7 month-old. Similar severe lesions were present in all examined striated skeletal muscles and were characterized by excessive fiber size variation with some large rounded hypertrophic fibers and clusters of small centronucleated regenerating fibers (**Fig. 3** and **Fig. S4**). Numerous individual necrotic fibers appeared fragmented or contained invading inflammatory cells (**Fig. 3** and **Fig. S4**), some being CD3-positive lymphocytes (data not shown). No calcium deposit was noticed after HES or Alizarin red stainings. ATPase staining revealed a predominance of type 1 fibers with perturbation of the normal random distribution of muscle fiber types, abnormal fiber grouping and presence of some type 2C fibers, described to be observed in case of a regenerating process after fiber necrosis (**Fig. S5**). These lesions were accompanied by a patchy increase in endomysial connective tissue at 3 months and a marked endomysial and perimysial fibrosis at 7 months (**Fig. 3 *A–C***). The main lesions observed in striated skeletal muscles were quantified by histomorphometry on the *biceps femoris* muscle. The mean minimal Ferret diameter was 41.1±20.9 µm *vs* 54.7±16.7 µm and 45.0±22.5 µm *vs* 54.7±18.5 µm, respectively at 3 and 7 months in *Dmd^mdx^* rat *versus* age-related controls (n = 5 in each group) showing a significant decrease in individual fiber size in *Dmd^mdx^* rats (p<0.0001) (**Fig. 3 *G, H***). This decreased diameter was illustrated by the modal value that was 30 to 40 µm in 3 and 7 month-old *Dmd^mdx^* rats (19.5±1.6% and 19.5±2.6%, respectively), whereas it corresponded to 50 to 60 µm in wild-type littermate controls (19.5±1.6% and 19.7±3.8%, respectively). The smallest fibers (with diameter less than 10 µm) represented 5.4±8.0% and 1.1±0.6% in 3 and 7 month-old *Dmd^mdx^* rats compared to the lower percentage in age-related control rats (0.5±0.7% and 0.0±0.0%, respectively). The strong regenerative activity suggested by this last result was assessed by examining expression of developmental myosin heavy chain isoform (MyoHC_Dev_), a marker specific to developing and regenerating myofibers. Abundant MyoHC_Dev_ positive fibers were observed in *biceps femoris* muscles of 3 and 7 month-old *Dmd^mdx^* rats *vs* control rats (13.1±6.7% and 10.2±4.4%, respectively, illustrating a slight decrease with time) and were absent in muscles of wild-type littermate controls (**Fig. 3 *D–F***). In addition, utrophin expression was found in fiber clusters with continuous membrane immunolabelling, also suggestive of regenerative processes as previously described in other models [28] (data not shown).

**Figure 4:**
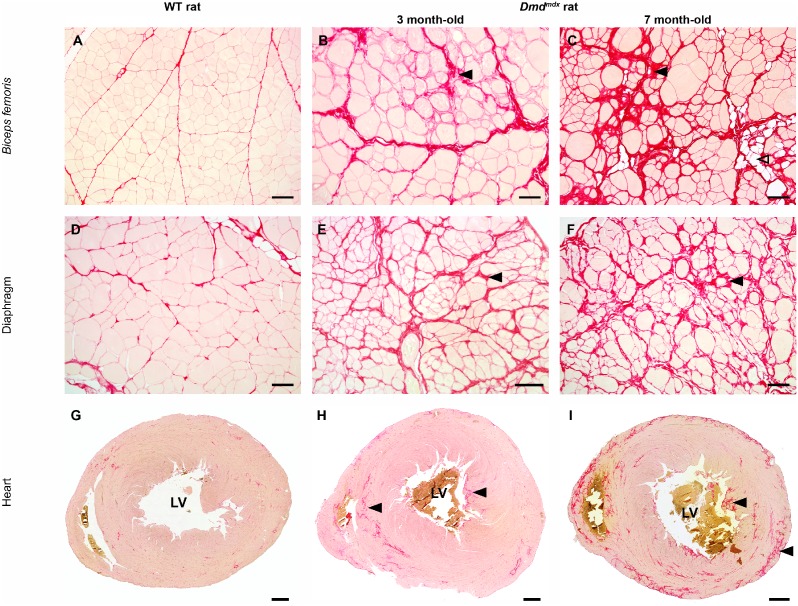
Progressive myofiber replacement by fibrotic and fat tissue in *Dmd^mdx^* rats. A picrosirius red staining specific for fibrosis was performed on *biceps femoris (A–C)*, respiratory (*D–F*) and heart muscle (*G–I*) samples obtained from wild-type littermate controls (WT) and 3 month-old and 7 month-old *Dmd^mdx^* rats. Compared to control rats (left panel), a progressive increase in the amount of fibrotic tissue (black arrowhead) was noticed in 3 (mid panel) and 7 month-old *Dmd^mdx^* rats (right panel). Note the focal presence of fat tissue infiltration (open arrowhead). In the heart, fibrosis was most marked in papillary muscle of the left ventricle (LV), in the septum and in the ventricular subepicardic area. Picrosirius red staining. Bar = 100 µm (A*–*F) and 1 mm (G*–*I).

The accumulation of connective tissue in *biceps femoris*, diaphragm and cardiac muscles was confirmed using a specific picrosirius staining in 7 month-old *Dmd^mdx^* rats and was associated mostly with muscle fiber focal replacement by adipose tissue (**Fig. 4**). In the heart, fibrosis was most prominent in papillary muscle of the left ventricle (**Fig. 4 *G–I***), in the septum and in the ventricular subepicardic area.

Total fibrosis was assessed by measuring the area occupied by connective tissue in transverse sections of *biceps femoris*. Connective tissue represented 16.9±4.7% and 24.3±8.7% in *Dmd^mdx^* rats compared to 6.7±3.2% and 6.4±2.3% in control rats at 3 and 7 months of age, respectively, demonstrating a significant effect of the mutation on total fibrosis (p<0.0001).

In the cardiac muscle, individual fiber necrosis associated with inflammatory cell infiltration and fibrosis predominated at 3 and 7 months in *Dmd^mdx^* rats (**Fig. S4 *C***). Fibrous connective tissue represented 8.0±4.9% and 15.7±7.9% of the total cardiac muscle area in *Dmd^mdx^* rats compared to 4.3±3.1% and 3.4±1.4% in wild-type littermate controls at 3 and 7 months, respectively. The increase in the fibrotic area from 3 to 7 months in *Dmd^mdx^* rats was significant (p<0.0001).

### *Dmd^mdx^* rats have a high level of serum creatine kinase

In DMD patients, muscle damage is characterized by an increase of creatine kinase (CK) activity in the serum. In *Dmd^mdx^* rats, CK levels showed a 10-fold augmentation when compared to wild-type littermate controls (CK (U.L^−1^). At 3 month-old, CK levels were 1945±620 in *Dmd^mdx^* rats (n = 5) and 185±17 in wild- type rats (n = 5) (p<0.05); At 7 month, these levels were 3965±817 in *Dmd^mdx^* rats (n = 5) and 588±397 in wild- type rats (n = 5) (p<0.05). These results suggest an increased fragility of muscle membrane and confirmed that *Dmd^mdx^* rats develop a dystrophic pathology.

### Significant muscle weakness in *Dmd^mdx^* rats

**Figure 5:**
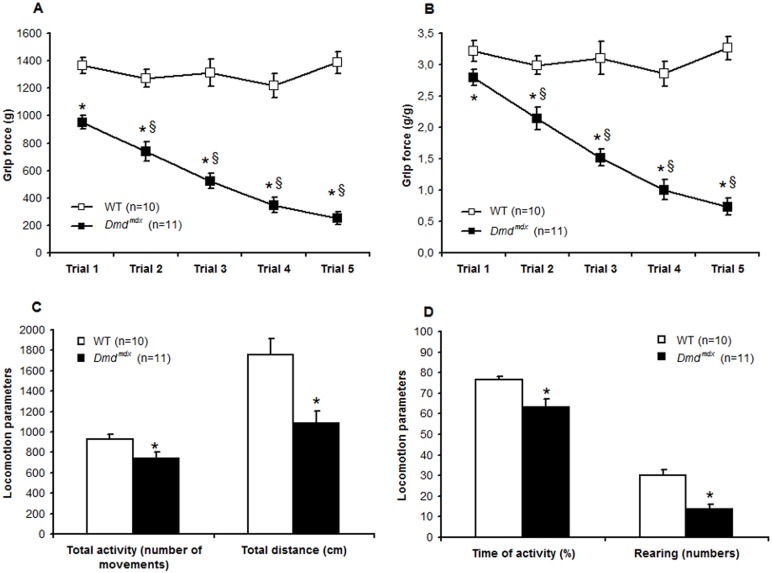
*Dmd^mdx^* rats are characterized by muscle weakness and by a decrease in a spontaneous activity. Forelimb grip test and locomotor activity of *Dmd^mdx^* rats were analyzed at the age of 3 months. Compared to wild-type littermate controls (WT), *Dmd^mdx^* rats were characterized by muscle weakness and showed a lower strength in absolute values (*A*) and when normalized to body weight (*B*). *Dmd^mdx^* rats showed fatigue with a decreased force grip across the five pulls, and significant values are obtained for the two last trials that were reduced to ∼70% when compared to Trial 1. At 3 months, when compared to WT, *Dmd^mdx^* rats were less active and the number of movements (*C*), the total distance travelled (*C*), the time of activity (*D*) and the number of rearing (*D*) were significantly lower. Values are means ± SEM; *p<0.05 *vs* WT; §: p<0.05 *vs* Trial 1.

To examine whether the absence of dystrophin affected muscle function, we performed a grip test on 3 month-old *Dmd^mdx^* rats. A significant decrease in forelimb grip strength indicated a generalized alteration in the whole body muscular performance. As illustrated in Fig. 5 (***A*** **, *B***), at the first trial to the grip a 30% weaker force was exerted by *Dmd^mdx^* rats compared to wild-type littermates. Furthermore, while control rats maintain the same force over five successive pulls, the grip force of *Dmd^mdx^* rats decreased significantly and became 70% weaker than that of control rats. Therefore, *Dmd^mdx^* rats presented a decreased grip strength test demonstrating muscle weakness, a typical sign observed in DMD patients [24], [29].

### Decrease in the spontaneous activity in *Dmd^mdx^* rats

Behavioral and locomotor measurements are important parameters that help to define the phenotypes of animal models with neuromuscular disorders such as muscular dystrophies [25]. These assessments were done in an open field plexiglass chamber equipped with multiple photocell receptors and emitters. At the age of 3 months, *Dmd^mdx^* rats were less active than control littermates and the number of movements, the total distance travelled and the time of activity were significantly lower respectively 21±7%; 38±7%; 17±5% lower in *Dmd^mdx^* than in wild-type littermate controls (p<0.05) (**Fig. 5** ***C, D***). Furthermore, the horizontal activity as rearing was also significantly impaired and reduced by 55±8% (p<0.05) in *Dmd^mdx^* rats compared to wild-type littermate controls (**Fig. 5** ***D***). These data demonstrate that motor activity and locomotor behavior are severely affected in *Dmd^mdx^* rats.

### Modification of the heart function in *Dmd^mdx^* rats

**Figure 6:**
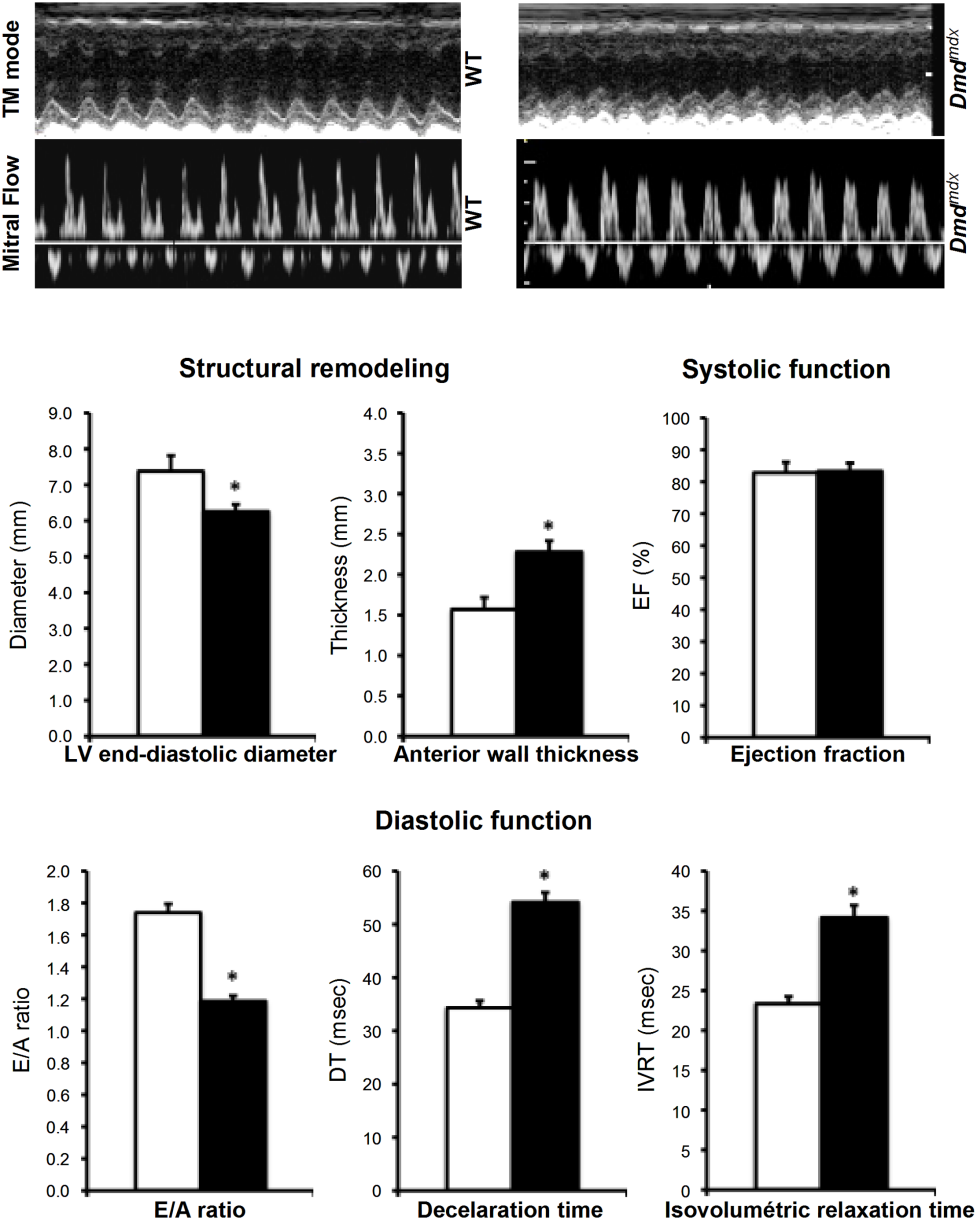
Structural remodelling and altered diastolic function in hearts of *Dmd^mdx^* rats. Echocardiography was performed on 3 month-old *Dmd^mdx^* rats. The structural remodeling and the systolic and diastolic functions were assessed respectively by Two-dimensional (2-D) echocardiography and pulsed Doppler (n = 6 for each condition); Values are mean ± SEM; *p<0.05). (TM mode: Time movement mode; LV: Left ventricular; E/A ratio: early diastolic (E)/late diastolic (A) ratio).

At 3 months, *Dmd^mdx^* rat and wild-type control littermates showed the same heart rate values under anaesthesia (HR_WT_ = 221±13 bpm and HR*_Dmd_^mdx^ = *219±12 bpm) (**Fig. 6**). The echocardiographic investigations revealed structural remodeling with a significant decrease in the LV end-diastolic diameter (EDD_WT_ = 7.4±0.4 mm and EDD*_Dmd_^mdx^ = *6.3±0.2 mm) associated with thickening of the LV end-diastolic anterior wall in *Dmd^mdx^* rats (EDAW_WT_ = 1.6±0.1 mm and EDAW*_Dmd_^mdx^ = *2.3±0.1 mm). These observations suggest the presence of a concentric remodeling in *Dmd^mdx^* rats heart at the age of 3 months. We did not observe any change in systolic function as ejection fraction (EF_WT_ = 82.3±3.3% and EF*_Dmd_^mdx^* = 83.3±2.6%) and shortening fractions (SF_WT_ = 44.3±4.4% and SF*_Dmd_^mdx^* = 47.8±2.8%; data not shown). However, diastolic dysfunction markers were observed. We observed changes in E/A ratio (E/A_WT_ = 1.7±0.1 and E/A*_Dmd_^mdx^* = 1.2±0.1), in Doppler–derived mitral deceleration time (DT_WT_ = 34.3±1.3 msec and DT*_Dmd_^mdx^* = 54.2±1.8 msec) and in isovolumetric relaxation time (IRT_WT_ = 23.3±0.9 msec and IRT*_Dmd_^mdx^* = 34.2±1.5 msec) (**Fig. 6**). These data demonstrate structural remodeling and altered diastolic function in hearts of *Dmd^mdx^* rats.

## Discussion

In this study, we describe the generation and the full characterization of a novel dystrophin deficient rat model (*Dmd^mdx^* rats) with phenotypic properties very close to the human DMD pathology.

DMD is the most common neuromuscular disorder, accounting for approximately 30% of muscular dystrophy patients [30]–[32]. The responsible gene is located in the short arm of the X chromosome, Xp21, and contains 14 kbp of dystrophin protein coding sequence split in 79 exons. The primary molecular characteristic of DMD patients is the absence of dystrophin protein in skeletal and cardiac muscles. The present results show that dystrophin was undetectable by western blot in skeletal muscles of *Dmd^mdx^* rats. In agreement, immunohistological analysis demonstrated that dystrophin is undetectable in 95% of fibers in skeletal muscles and only some revertant fibers were visible, as in DMD patients and other animal models of DMD [27]. Immunostaining and western blot analyses demonstrated that dystrophin was also undetectable in hearts of *Dmd^mdx^* rats. The absence of dystrophin, as encountered in DMD patients, leads to absence of the dystro-associated protein complex, instability of the muscle cell membrane and uncontrolled influx of calcium. This initiates a set of downstream pathological processes that ultimately lead to loss of muscle cell proteins, myofiber damage and progressive muscle wasting [33]. Similar to muscle damages in DMD patients, *Dmd^mdx^* rats are characterized by a loss of skeletal muscle mass, muscle fiber necrosis, increased variation in fiber size due to the simultaneous presence of hypertrophic fibers and small centronucleated regenerating fibers, infiltration by inflammatory cells, regenerative activity and interstitial fibrosis in skeletal muscles. All these signs of tissue changes observed in limb and forelimb skeletal muscles are correlated with a dramatic increase of serum creatine kinase activity in 3 and 7 month-old *Dmd^mdx^* rats. As observed in DMD patients but not in *mdx* mice [34], fibrosis was severe in all skeletal muscle examined as well as in cardiac muscle of *Dmd^mdx^* rats (**Fig. 4**). It is well described in dystrophin deficient skeletal myofibers that Ca^2+^ homeostasis alteration is pivotal in the initiation of the fiber damages [35], [36]. In skeletal muscle from *Dmd^mdx^* rats at 3 and 7 months, while severe lesions were present no calcification was observed. Thus it will be interesting to further investigate calcium regulation in *Dmd^mdx^* rat skeletal muscles.

Consistent with histomorphological muscle damages, at 3 and 7 months of age, *Dmd^mdx^* rats exhibit muscle dysfunction with decrease in muscle force and reduced locomotor activity. The clinical manifestations of *Dmd^mdx^* rats evaluated at 3 and 7 months of age were progressive with gradual loss of body weight and muscle mass (mainly of fast twitch muscles such as *tibialis anterior*) as well as in necrosis and degeneration of skeletal muscle fibers including fundamental muscles such as the diaphragm. Additionally, *Dmd^mdx^* rats showed a progressive dilated cardiomyopathy as indicated by morphological analyses, concentric structural remodeling and diastolic dysfunction. Since the majority of DMD patients die from cardiac failure [37] the cardiac dysfunction described in *Dmd^mdx^* rats is an important feature of this new DMD model.

All skeletal and cardiac lesions as well as functional deficits are important features of the rat *Dmd^mdx^* model since they are similar to those of patients. It will be interesting to study the disease onset in this new model as was previously studied in the *mdx* mouse, in particular to identify the early prenecrotic stages. Importantly, the changes observed between the two time points of this study i.e 3 and 7 month-old showed a progressive evolution of the pathology in this rat model, in the same manner than those described in DMD patients. Therefore, *Dmd^mdx^* rats will be useful for pre-clinical assessment of new therapies. In particular, as analyses of dystrophin mRNA in the muscles of *Dmd^mdx^* rats showed that the mutated exon 23 was transcribed (**Fig. S6**). *Dmd^mdx^* rats could therefore be used for the evaluation of DMD treatment using an exon skipping approaches.

The use of TALENs targeting the rat *Dmd* gene was efficient at introducing *Dmd* mutations. Other types of gene-specific nucleases are available, such as ZFNs and more recently CRISPRs/Cas9, that have also been used successfully in rats [9]–[13], [38]. Therefore, the generation of new rat models of neuromuscular disease using artificial sequence-specific nucleases now appears very promising. While this manuscript was in preparation, dystrophin-deficient rats generated with CRISPR/Cas9 targeting exon 2 and exon 16 were reported [38]. The phenotype of the corresponding F0 rats showed characteristics similar to the *Dmd^mdx^* rats in skeletal muscle although progression of the disease was not documented. In the heart, in contrast, even though morphological changes were reported in some 3-month old F0 animals, they were not found statistically significant. The difference between the two models could be due to differences in genetic backgrounds (Wistar-Imamichi inbred *vs* Sprague-Dawley outbred strain for *Dmd^mdx^*) or to mosaicism of *Dmd* mutations in F0 animals studied in the latter report.

The genetic heterogeneity and body size of the Golden Retriever Muscular Dystrophy dog model (GRMD) is closer to humans than the mice (**Table 2**). Therefore, GRMD is an important animal model for DMD. However, one major disadvantage is the large heterogeneity in the clinical phenotypes of GRMD dogs that makes the functional evaluation of potential therapies difficult. The *mdx* mouse has been important as a DMD model for development of gene therapy approaches. Murine models as *mdx*, *mdx* ^5cv^, *mdx*52 will continue to provide important findings for the basic study of pathogenesis and development of therapies but they do not show significant muscle weakness or cardiac alterations nor any of the skeletal or cardiac muscle lesions developing at late time points in DMD patients, such as fibrosis and muscular atrophy. In contrast, it was shown that *mdx*/mTR^KO^ mice, combining the *mdx* mutation with deletion of the gene for the RNA component of telomerase, show skeletal muscle dysfunction and progressive dilated cardiomyopathy as a result of shortened telomers and accelerated depletion of stem cell reservoirs [39], [40]. Given the much aggravated skeletal and cardiac muscle phenotype of *Dmd^mdx^* rats compared to *mdx* mice, it will be interesting to look for any difference in telomere dynamics or stem cell biology between these two closely related species that could be involved.

**Table 2:** Comparison of pathological and functional characteristics between patients and animal models of Duchenne disease.

	DMD patient	GRMD dog	DMD pig	HFMD cat	Mdx mouse	*Dmd^mdx^* rat
**Muscle histopathology**						
Revertant fibers	1 to 3%	<1%	ND	ND	5%	**5%**
Necrotic fibers	0.5 to 3.5%	2%	absent	present	5%	**>10%**
Regeneration	ND	15%	ND	present	10%	**10%**
Calcification	mild	mild tomarked	ND	severe	mild	**absent**
Fibrosis	marked	present	present	diaphragmmainly	late & mainlydiaphragm	**marked**
Lipomatosis	severe	absent	absent	absent	absent	**mild**
Cardiomyopathy	marked, majorcause of death	mild	absent	present	absent or late	**marked**
**Muscle function**						
Strength reduction	marked	marked	ND	ND	mild	**marked**
Locomotion	severely impaired	impaired	impaired	ND	normal	**impaired**

In summary, our study shows that *Dmd^mdx^* rats represent a promising model to further elucidate mechanisms of DMD and to test novel therapies, in particular those aiming to curtail heart alterations and skeletal muscle disease progression.

## Supporting Information

Figure S1 **Mutation rates of TALE nucleases targeting exon 23 of the rat** ***Dmd*** **gene.** The mutation rates induced by TALE nuclease were determined using T7 endonuclease assay in C6 cells transfected with indicated amounts of rat *Dmd* TALE nucleases expression vectors. Homodimeric (WT, 0.75 µg each) and heterodimeric (EDL/KKR; 0.75 µg and 1.5 µg each) TALEN pairs (DMD-L+DMD-R) have been evaluated as well as left and right part of the TALEN pair (1.5 µg). The expected sizes of digested fragments are indicated near the gel. The rates of insertion and deletion mutations (indels) detected are indicated below each lane. The TALEN binding sites in exon 23 are indicated in blue.
(TIFF)

Figure S2 **Growth of** ***Dmd^mdx^*** **rats.** Newborns at day 2 (*A*). While wild-type littermate controls (WT) and *Dmd^mdx^* rats were indistinguishable after birth, *Dmd^mdx^* rats were noticeably smaller at the age of 16 weeks (*B*). Body weight values from WT and *Dmd^mdx^* rats from 4 to 12 weeks postnatal (*C*). Values are mean ± SEM; *p<0.05 *Dmd^mdx^ vs* WT, analyzed by unpaired *t* test.
(TIF)

Figure S3 **The absence of dystrophin in skeletal muscle fibers is associated with a strong reduction in alpha-sarcoglycan expression in** ***Dmd^mdx^*** **rats.** *Biceps femoris* muscle samples were assessed for the expression of proteins of the dystrophin associated protein complex by immunohistochemistry. Compared to the generalized subsarcolemmal expression of alpha-sarcoglycan in wild-type littermate control (WT) rats (*A*), only some fibers expressed the protein in 7 month-old *Dmd^mdx^* rats (*B*). Bar = 100 µm.
(TIF)

Figure S4 **Muscle changes were present in all striated muscle including skeletal, respiratory and cardiac ones in** ***Dmd^mdx^*** **rats at 7 month-old.** In *extensor digitorum longus* (*A*) and diaphragm (*B*) muscles, hypercontracted hyalin giant fibers (°) and individual necrotic fibers (open arrowhead), sometimes surrounded by some inflammatory cells (#), were associated with foci of small regenerative centro-nucleated fibers (arrow). Some muscle fibers were replaced by fibrotic (*) and fat (black arrowhead) tissue. In cardiac muscle (*C*), individual fiber necrosis (open arrowhead) elicited some inflammatory cell infiltration (#). Focal thick bundles of fibrotic tissue surround cardiomyocytes (*). Hemalun eosin saffron staining (*A–C*). Bar = 100 µm.
(TIF)

Figure S5 **Abnormal fiber type pattern in** ***Dmd^mdx^*** **rats.** Compared to wild-type littermate control (WT) rats, *biceps femoris* muscles from 7-month-old *Dmd^mdx^* rats were characterized by type 1 predominance and grouping (type 1 fibers in black) and abnormal presence of type 2C (intermediate grey staining, open arrowhead). ATPase staining, pH 4.35. Bar = 100 µm.
(TIF)

Figure S6 **Dystrophin messenger RNA expression in skeletal muscles and hearts of** ***Dmd^mdx^*** **rats.** Dystrophin mRNA was detected by nested RT-PCR from exon 22 to 24 in *tibialis cranialis* muscles and in hearts sampled in 7 month-old *Dmd^mdx^* rats and wild-type control littermates. No skipping of the mutated exon 23 was detected in muscles from *Dmd^mdx^* rats.
(TIF)

Table S1 **Efficacy of generation of** ***Dmd*** **mutants by TALE nuclease microinjection.**
(DOC)

Table S2 ***Dmd*** **mutations and potential off target sequences analyzed in founder animals.**
(DOC)
