# Supplementary figures and images for: Characterization of Dystrophin Deficient Rats: A New Model for Duchenne Muscular Dystrophy
Source: PLoS One. 2014 Oct 13;9(10):e110371. doi: 10.1371/journal.pone.0110371 (PMC4195719; doi:10.1371/journal.pone.0110371)

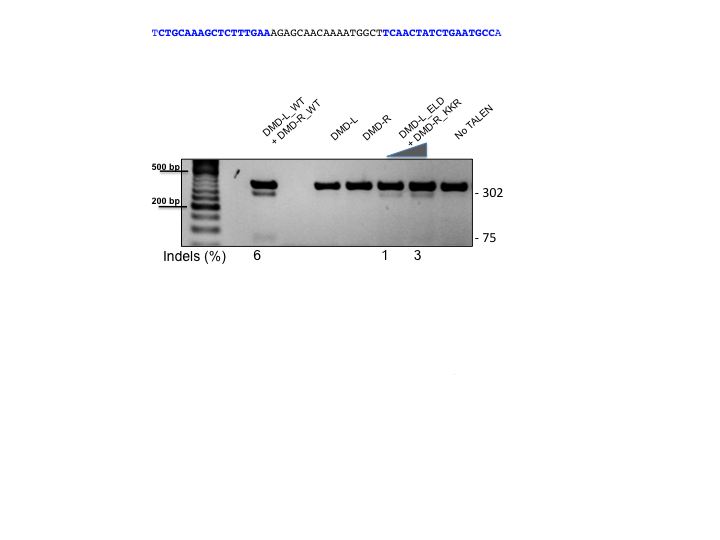

Supplement: Figure S1 — Mutation rates of TALE nucleases targeting exon 23 of the rat Dmd gene. The mutation rates induced by TALE nuclease were determined using T7 endonuclease assay in C6 cells transfected with indicated amounts of rat Dmd TALE nucleases expression vectors. Homodimeric (WT, 0.75 µg each) and heterodimeric (EDL/KKR; 0.75 µg and 1.5 µg each) TALEN pairs (DMD-L+DMD-R) have been evaluated as well as left and right part of the TALEN pair (1.5 µg). The expected sizes of digested fragments are indicated near the gel. The rates of insertion and deletion mutations (indels) detected are indicated below each lane. The TALEN binding sites in exon 23 are indicated in blue. (TIFF) [file pone.0110371.s001.tiff]

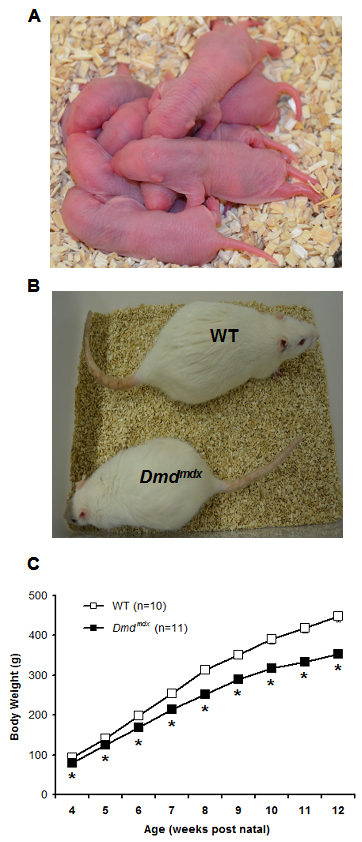

Supplement: Figure S2 — Growth of Dmdmdx rats. Newborns at day 2 (A). While wild-type littermate controls (WT) and Dmdmdx rats were indistinguishable after birth, Dmdmdx rats were noticeably smaller at the age of 16 weeks (B). Body weight values from WT and Dmdmdx rats from 4 to 12 weeks postnatal (C). Values are mean ± SEM; *p<0.05 Dmdmdx vs WT, analyzed by unpaired t test. (TIF) [file pone.0110371.s002.tif]

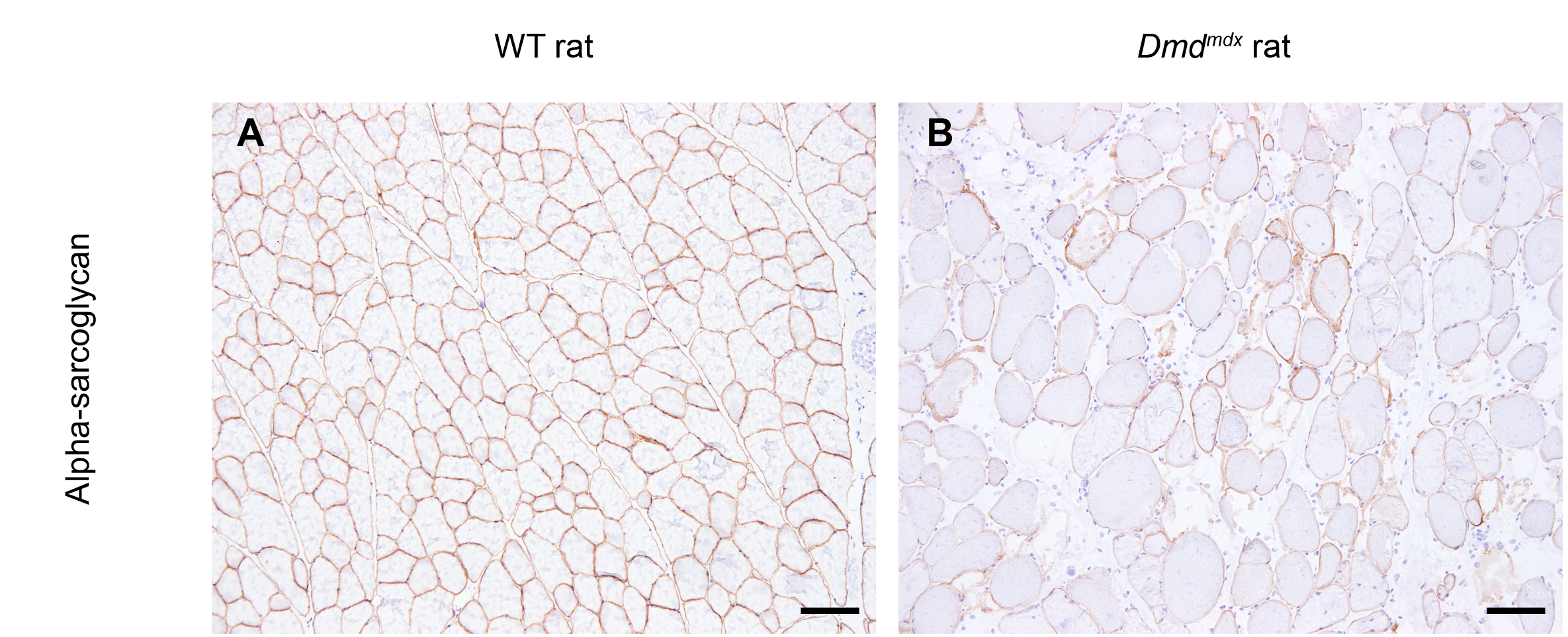

Supplement: Figure S3 — The absence of dystrophin in skeletal muscle fibers is associated with a strong reduction in alpha-sarcoglycan expression in Dmdmdx rats. Biceps femoris muscle samples were assessed for the expression of proteins of the dystrophin associated protein complex by immunohistochemistry. Compared to the generalized subsarcolemmal expression of alpha-sarcoglycan in wild-type littermate control (WT) rats (A), only some fibers expressed the protein in 7 month-old Dmdmdx rats (B). Bar = 100 µm. (TIF) [file pone.0110371.s003.tif]

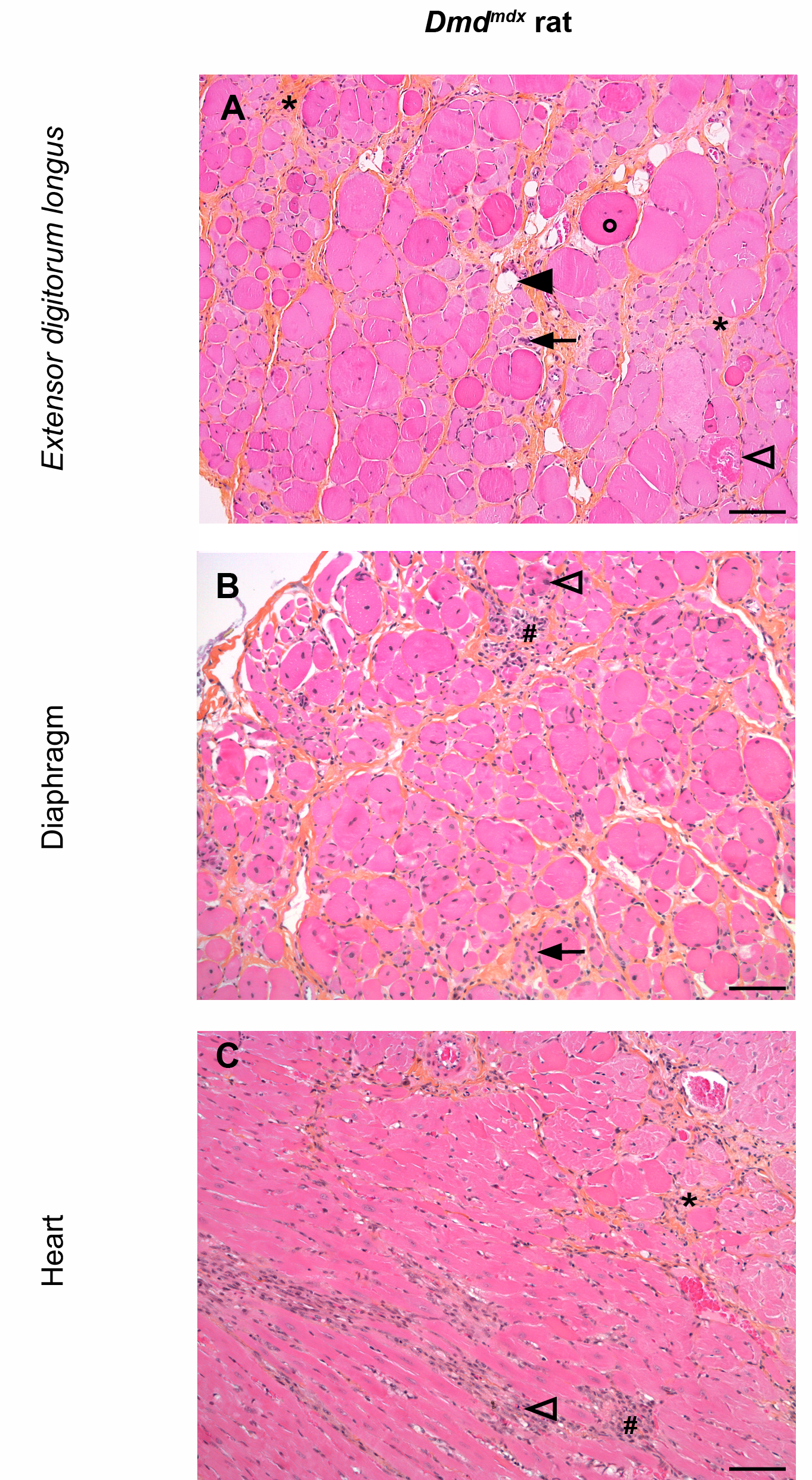

Supplement: Figure S4 — Muscle changes were present in all striated muscle including skeletal, respiratory and cardiac ones in Dmdmdx rats at 7 month-old. In extensor digitorum longus (A) and diaphragm (B) muscles, hypercontracted hyalin giant fibers (°) and individual necrotic fibers (open arrowhead), sometimes surrounded by some inflammatory cells (#), were associated with foci of small regenerative centro-nucleated fibers (arrow). Some muscle fibers were replaced by fibrotic (*) and fat (black arrowhead) tissue. In cardiac muscle (C), individual fiber necrosis (open arrowhead) elicited some inflammatory cell infiltration (#). Focal thick bundles of fibrotic tissue surround cardiomyocytes (*). Hemalun eosin saffron staining (A–C). Bar = 100 µm. (TIF) [file pone.0110371.s004.tif]

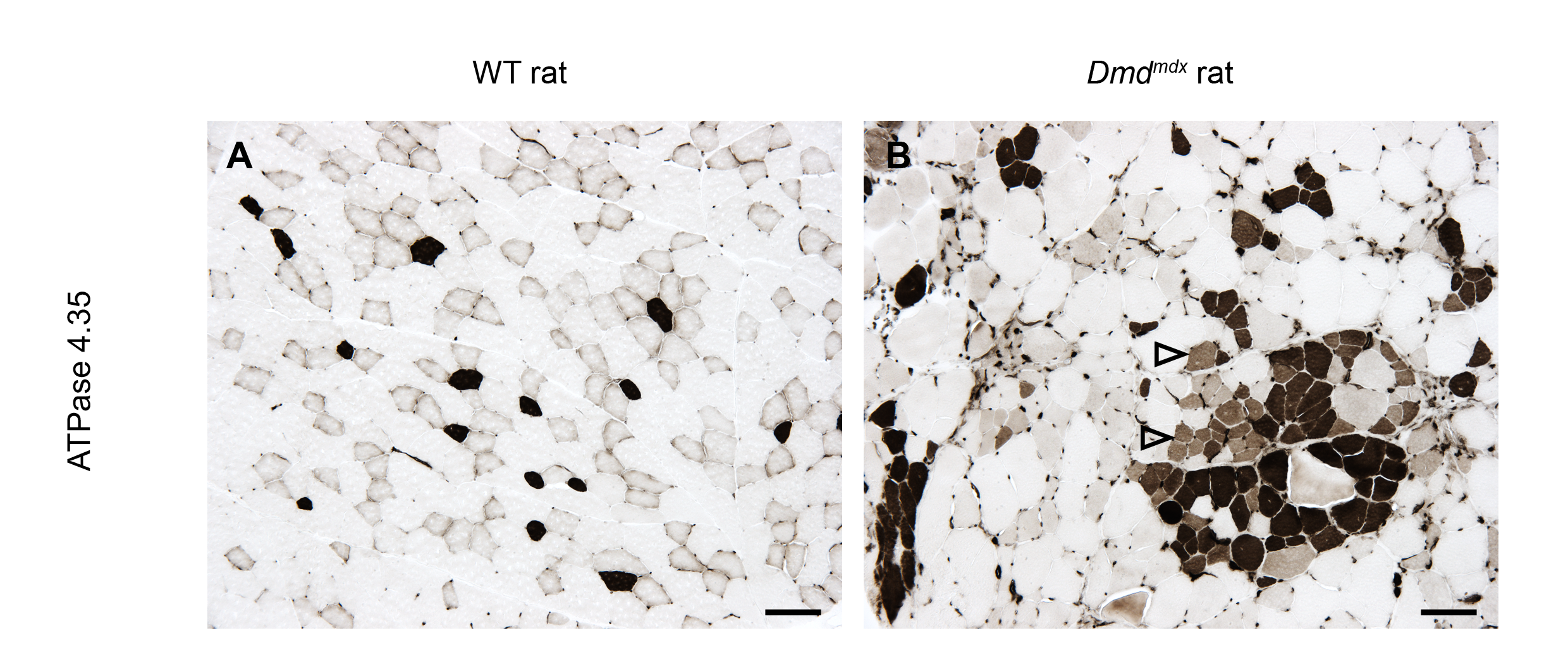

Supplement: Figure S5 — Abnormal fiber type pattern in Dmdmdx rats. Compared to wild-type littermate control (WT) rats, biceps femoris muscles from 7-month-old Dmdmdx rats were characterized by type 1 predominance and grouping (type 1 fibers in black) and abnormal presence of type 2C (intermediate grey staining, open arrowhead). ATPase staining, pH 4.35. Bar = 100 µm. (TIF) [file pone.0110371.s005.tif]

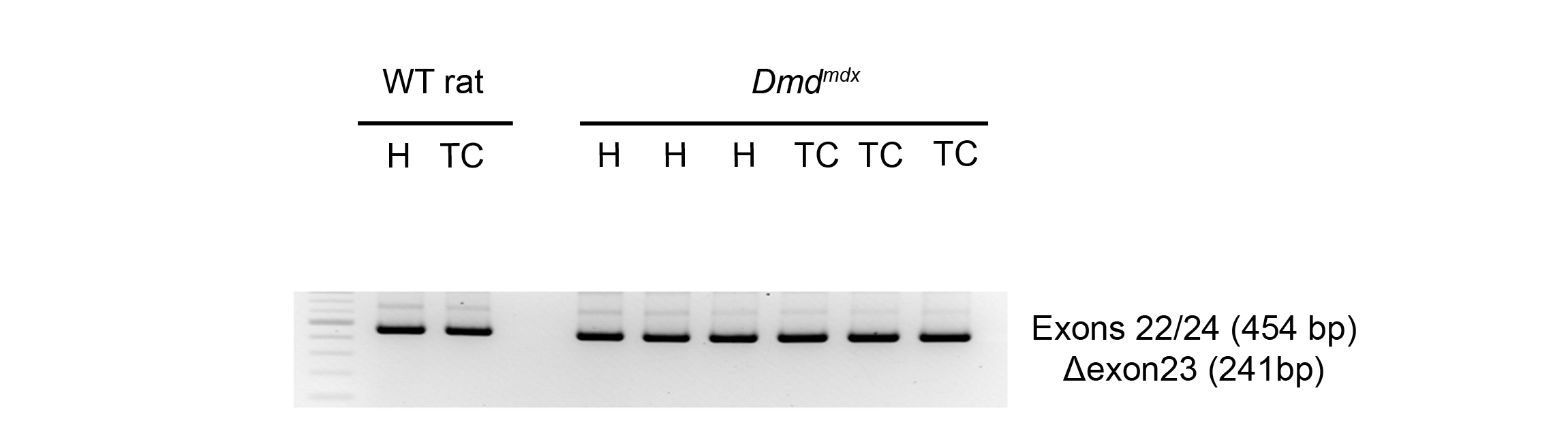

Supplement: Figure S6 — Dystrophin messenger RNA expression in skeletal muscles and hearts of Dmdmdx rats. Dystrophin mRNA was detected by nested RT-PCR from exon 22 to 24 in tibialis cranialis muscles and in hearts sampled in 7 month-old Dmdmdx rats and wild-type control littermates. No skipping of the mutated exon 23 was detected in muscles from Dmdmdx rats. (TIF) [file pone.0110371.s006.tif]
