# Supplementary material for: Characterization of Dystrophin Deficient Rats: A New Model for Duchenne Muscular Dystrophy
Source: PLoS One. 2014 Oct 13;9(10):e110371. doi: 10.1371/journal.pone.0110371 (PMC4195719; doi:10.1371/journal.pone.0110371)
Supplement: Table S1 — Efficacy of generation of Dmd mutants by TALE nuclease microinjection. (DOC) [file pone.0110371.s007.doc]

**Table S1.** Efficacy of generation of *Dmd* mutants by TALE nuclease microinjection.

| n° injected  embryos | n° viables embryos  (% microinjected) | n° transferred embryos | n° newborns  (% transferred) | n° mutants  (% transferred embryos) |
| --- | --- | --- | --- | --- |
| 387 | 320  (82.7) | 294* | 88**  (30) | 11***  (3.74) |

*13 transfers ; **10 deliveries ; *** 4 females and 7 males
