# Supplementary material for: Characterization of Dystrophin Deficient Rats: A New Model for Duchenne Muscular Dystrophy
Source: PLoS One. 2014 Oct 13;9(10):e110371. doi: 10.1371/journal.pone.0110371 (PMC4195719; doi:10.1371/journal.pone.0110371)
Supplement: Table S2 — Dmd mutations and potential off target sequences analyzed in founder animals. (DOC) [file pone.0110371.s008.doc]

**Table S2. *Dmd* mutations and potential off target sequences analyzed in founder animals.**

**Wild type**

2886- CTG CAA AGC TCT TTG AAA GAG CAA CAA AAT GGC TTC AAC TAT CTG AAT GCC - **STOP at 10965 bp**

L Q S S L K E Q Q N G F N Y L N

**Male 9** (**deletion 16 bp + insertion 3 bp**)

2886-CTG CAA AGC TCT TTG AAA xxx xxx xxx xxxx cta TCA ACT ATC **TGA** ATG CC- - **STOP at 9 bp, small litters**

L Q S S L K L S T I

**Male 23** / **Female 60** / **Male 71** / **Male 80 (same 1bp deletion)**

2886-CTG CAA AGC TCT TTG AAA GAG CAA CAA AxTG GCT TCA ACT ATC **TGA** ATG CC - - - - -**STOP at 14 bp**

L Q S S L K E Q Q M A S T I

**Male 44 / Male 61** (**deletion 11 bp)**

2886-CTG CAA AGC TCT TTG AAA GAG xxx xxx xxx xxCTT CAA CTA TCT GAA TGC C - - - - **STOP at 81 bp**

L Q S S L K E L Q L S E …………….

**Female 51** (**deletion 7 bp, the other allele wild type**)

2886-CTG CAA AGC TCT TTG AAA GAG xxx xxx xCTG GCT TCA ACT ATC **TGA** ATG CC- -**STOP at 15 bp, small litters**

L Q S S L K E L A S T I

**Female 56 (one allele deletion 11 bp and the other 24 bp)**

2886-CTG CAA AGC TCT TTG AAA xxx xxx xxx xx TGG CTT CAA CTA TCT GAA TGC- - - **STOP at 84 bp**

L Q S S L K W L Q L S E

2886-CTG CAA AGC xxx xxx xxx xxx xxx xxx xxx xxx TTC AAC TAT CTG AAT - - - -

L Q S F N Y L N  **∆ 8 aa,** **no frame shift**

**Female 76 (deletion 12 bp, the other allele wild type)**

2886-CTG CAA AGC TCT TTG xxx xxx xxx xxx AAT GGC TTC AAC TAT CTG AAT

L Q S S L N G F N Y L N  **∆ 4 aa, no frame shift**

**Male 82** (**insertion 1 bp**)

2886-CTG CAA AGC TCT TTG AAA GAG CAA CAA AAa TGG - - - - **STOP at 87 bp, no offspring**

L Q S S L K E Q Q K W +28aa

**Potential off–target sequences analyzed:**

**Sequence** chrX:111,159,013-111,159,074 **(rn5) located in intron of** collagen type IV alpha 6 precursor gene  :

TttCATTCAGcTAtTTGAAATGGGAAGACAGCACACTGATCCATTTttAAGAGgTTTGCAGc

**Sequence chrX:129,901,352-129,901,414 (rn5) located in intergenic region:**

TCTatAAcGCcCTTTaAAAATGGAATAAGATCCTTTGCAAGTGATTtAcCTATaTGAATGtgA

___________________________________________________________________________________________

Underlined sequence correspond to DNA-binding sites of TALE nuclease monomer or remaining portions thereof following mutagenesis; Deletions=x; Insertions=small characters; boxed TGA=STOP codon. Underlined are lines 61, fully described in this manuscript, and 71 with similar phenotype and functional defects and with data not shown. In off-target sequences analyzed, potential TALEN binding sequences are underlined with mismatches to the TALEN target sequence in the DMD gene in lowercase. No mutations could be detected at candidate off-target sites from the X-chromosome in tail DNA from 6 F0 (including the founder rats #61 and #71) and 8 F2 rats derived from founder rat #61 (including 4 DMD mutant animals) using the T7 endonuclease 1 assay (data not shown).
